# Temporal and spatial analysis of *Plasmodium falciparum* genomics reveals patterns of parasite connectivity in a low-transmission district in Southern Province, Zambia

**DOI:** 10.1186/s12936-023-04637-9

**Published:** 2023-07-07

**Authors:** Abebe A. Fola, Kara A. Moser, Ozkan Aydemir, Chris Hennelly, Tamaki Kobayashi, Timothy Shields, Harry Hamapumbu, Michael Musonda, Ben Katowa, Japhet Matoba, Jennifer C. Stevenson, Douglas E. Norris, Philip E. Thuma, Amy Wesolowski, William J. Moss, Jeffrey A. Bailey, Jonathan J. Juliano, the Southern, Central Africa International Center of Excellence for Malaria Research (ICEMR)

**Affiliations:** 1 Department of Pathology and Laboratory Medicine, Brown University, 55 Claverick Street, Providence, RI 02906 USA; 2 University of North Carolina Institute for Global Health and Infectious Diseases, University of North Carolina Chapel Hill, Chapel Hill, NC 27599 USA; 3 Department of Epidemiology, Johns Hopkins Bloomberg School of Public Health, Baltimore, MD 21205 USA; 4 Macha Research Trust, Choma District, Choma, Zambia; 5 Department of Molecular Microbiology and Immunology, The Johns Hopkins Malaria Research Institute, Johns Hopkins Bloomberg School of Public Health, Baltimore, MD 21205 USA; 6 Division of Infectious Diseases, School of Medicine, University of North Carolina Chapel Hill, Chapel Hill, NC 27599 USA; 7 Department of Epidemiology, Gillings School of Global Public Health, University of North Carolina Chapel Hill, Chapel Hill, NC 27599 USA; 8 Curriculum in Genetics and Molecular Biology, School of Medicine, University of North Carolina Chapel Hill, Chapel Hill, NC 27599 USA

**Keywords:** *Plasmodium falciparum*, Transmission, Zambia, Genomics

## Abstract

**Background:**

Understanding temporal and spatial dynamics of malaria transmission will help to inform effective interventions and strategies in regions approaching elimination. Parasite genomics are increasingly used to monitor epidemiologic trends, including assessing residual transmission across seasons and importation of malaria into these regions.

**Methods:**

In a low and seasonal transmission setting of southern Zambia, a total of 441 *Plasmodium falciparum* samples collected from 8 neighbouring health centres between 2012 and 2018 were genotyped using molecular inversion probes (MIPs n = 1793) targeting a total of 1832 neutral and geographically informative SNPs distributed across the parasite genome. After filtering for quality and missingness, 302 samples and 1410 SNPs were retained and used for downstream population genomic analyses.

**Results:**

The analyses revealed most (67%, n = 202) infections harboured one clone (monogenomic) with some variation at local level suggesting low, but heterogenous malaria transmission. Relatedness identity-by-descent (IBD) analysis revealed variable distribution of IBD segments across the genome and 6% of pairs were highly-related (IBD ≥ 0.25). Some of the highly-related parasite populations persisted across multiple seasons, suggesting that persistence of malaria in this low-transmission region is fueled by parasites “seeding” across the dry season. For recent years, clusters of clonal parasites were identified that were dissimilar to the general parasite population, suggesting parasite populations were increasingly fragmented at small spatial scales due to intensified control efforts. Clustering analysis using PCA and t-SNE showed a lack of substantial parasite population structure.

**Conclusion:**

Leveraging both genomic and epidemiological data provided comprehensive picture of fluctuations in parasite populations in this pre-elimination setting of southern Zambia over 7 years.

**Supplementary Information:**

The online version contains supplementary material available at 10.1186/s12936-023-04637-9.

## Background

As malaria elimination efforts continue to drive down disease burden in parts of Africa, some regions previously endemic for malaria have seen drastic reductions in overall morbidity and mortality [1, 2]. In these areas approaching pre-elimination, identification of ongoing local transmission and importation events is critical for maintaining elimination gains and preventing outbreaks in increasingly susceptible populations. Identifying reservoirs for continued transmission, which may occur in specific geographical locations, temporal periods, or age groups, would allow targeted elimination efforts to interrupt sustained transmission. Identifying suspected imported cases (where a genetically distinct parasite is introduced to a region) would assist malaria control programmes in identifying high-trafficked routes of human movement and sources of importation [3–5]. Blocking such sources and identifying corridors for parasite importation would also be key to protect against introductions to populations increasingly more susceptible to malaria after successful control efforts [6, 7].

**Fig. 1:**
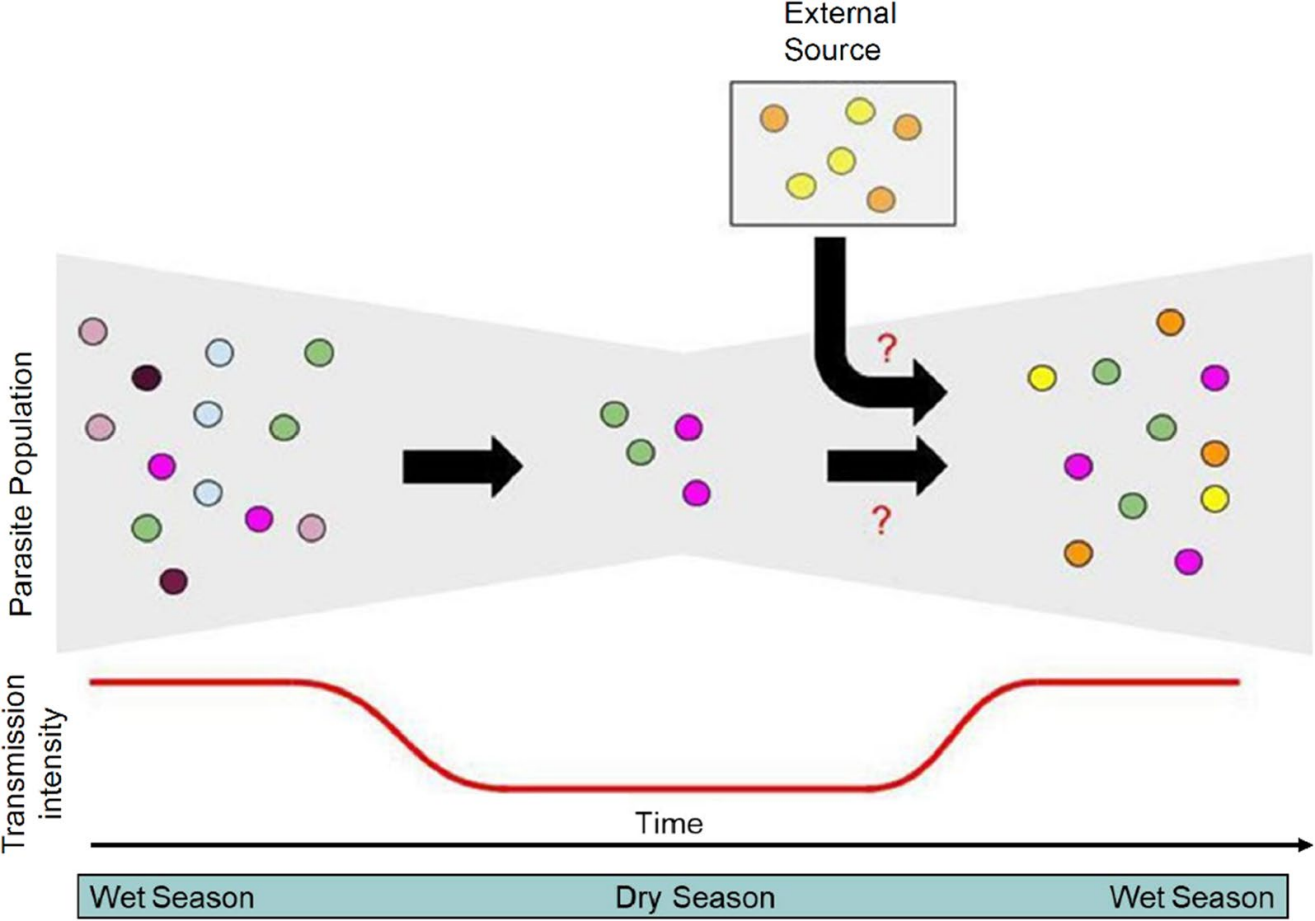
Theoretical construct for continued transmission in low transmission settings. Malaria transmission in a low endemicity setting is likely the result of a combination of persistence through the dry, low transmission season and importation from other regions. However, the relative contribution of these remains unknown (red question marks). Parasite genomics can help understand these relationships. Different parasite lineages (colours of circles) may or may not survive through a dry season, shown by the reduction in diversity during the dry season. However, genetic diversity may be enhanced through importation through an external source

Parasite genomic epidemiology can be used to track transmission patterns in pre-elimination settings and help elucidate the mechanisms of persistent transmission (Fig. 1). Following decreases in pathogen transmission, it is expected that parasite population sizes will also shrink, reflected in reduced genetic variation fueled by higher levels of clonal transmission and inbreeding, fracturing population structure [8–10]. These genetic patterns should follow reductions in disease burden over the long-term but may also be seen in areas with distinct seasonality with low transmission periods (e.g. dry seasons). These expectations of low genetic variation in association with low transmission may not hold if an area receives high numbers of imported cases [11, 12]. Additionally, these regions may be more sensitive to temporal fluctuations of malaria cases and parasite populations, as increased transmission and outbreaks might be more common due to decreasing immunity [13].

These low transmission settings offer a unique opportunity to observe long-term dynamics of parasites across seasons, as the smaller number of parasites may be easier to track because of a lack of recombination with genetically distinct parasites within the mosquito. A small number of studies have attempted to observe temporal population changes in low transmission settings, and even fewer have been able to combine temporal and geospatial data to identify hotspots over space and time [3, 8, 11, 14–17]. Most of these studies evaluated parasite population changes over relatively large geographic distances. Thus, it remains unknown if and how parasite populations change over time in low transmission settings on a small spatial scale, such as a district.

Southern Zambia has had a drastic reduction in the number of malaria cases over the past two decades [1]. Despite this reduction, Choma District in Southern Province, Zambia consistently has had a low number of malaria cases every year that occur seasonally [18]. However, it is not understood how the parasites driving this low-level transmission are maintained between seasons. The transmission dynamics that connect falciparum malaria infections across space and time in Choma District, Southern Province, Zambia from 2012 to 2018 were characterized using a high-throughput genomic tool, molecular inversion probes (MIPs). This approach allows us to develop a more refined understanding of transmission dynamics and parasite population connectivity than previous studies using low resolution genotyping as identity-by-descent (IBD) approaches can be used to track relatedness between parasite isolates. IBD has been shown to be superior for understanding the relatedness and interconnectivity of parasite populations [19–21]. The results showed that highly related parasites are connected across multiple seasons, suggesting that cases are at least in part fueled by parasites persisting through the dry season. In addition, two different clusters of clonal parasites that are distinct from the general parasite population were identified, suggesting intensified control efforts have led to fracturing the parasite population at the local level and the success of malaria control at the subnational level.

## Methods

### Sample collection and MIP sequencing

Samples were collected through passive case detection from eight health centres in and around the catchment area of Macha Hospital in Choma District, Southern Province, Zambia in an area of approximately 2000 km^2^. This work was approved as part of Southern and Central Africa International Center of Excellence for Malaria Research by the Tropical Diseases Research Center, Ndola, Zambia (Ref No: TDRC/ERC/2010/14/11) and the Johns Hopkins Bloomberg School of Public Health (IRB #3467). Analyses utilizing parasite genomes from de-identified samples were deemed nonhuman subjects of research at the University of North Carolina at Chapel Hill (NC, USA) and Brown University (RI, USA).

From 2012 to 2018, dried blood spots (DBS) were collected from 441 symptomatic individuals presenting to the health centres who were positive for *Plasmodium falciparum* infection by rapid diagnostic test. DNA was extracted from each DBS with a Chelex-Tween protocol [22]. Parasitaemia was assessed using quantitative PCR with probes targeting the *pfldh* gene [23]. Samples were then genotyped using molecular inversion probes (MIPs) targeting 1834 neutral and geographical informative SNPs distributed across the *P. falciparum* nuclear genome [24, 25]. MIP capture and library preparation were done as previously described [24]. The MIP library was sequenced in two sequencing runs using an Illumina NextSeq 550 instrument (150 bp paired-end reads) at Brown University (RI, USA).

### Bioinformatic analysis

Processing of sequencing data and variant calling was done using MIPtools (v0.19.12.13; https://github.com/bailey-lab/MIPTools), a suite of computational tools designed to process sequencing data from MIPs. Raw reads from each MIP, identifiable using unique molecular identifiers (UMIs), were used to reconstruct sequences using MIPWrangler, and variant calling was performed on these samples using freebayes [26]. To reduce false positives due to PCR and alignment errors, the alternative allele (SNP) must have been supported by more than one UMI within a sample, and the allele must have been represented by at least 10 UMIs across the entire population. Biallelic, variant SNP positions were retained for downstream analyses. Moreover, individual variant calls within each sample were set to be missing if the site was not supported by at least four UMIs. After these steps, genome positions (SNPs) with more than 50% missing data, followed by samples with less than 50% data, were removed from all downstream analyses. Variants were annotated using the 3D7 v3 reference genome.

### Population genetic analyses

Using the final variant set (n = 1410 SNPs) distributed across the genome (Additional file 1: Fig. S1), the complexity of infection (COI) for each sample was determined using THE REAL McCOIL categorical method [27] which turns heterozygous SNP data into robust estimates of allele frequency, via Markov chain Monte Carlo (MCMC) methods.

Genetic relatedness of sample -pairs was assessed using the major allele at each position to estimate inbreeding coefficients, calculated using a maximum-likelihood approach that estimates the probability that any position is identical-by-descent between two samples using MIP analyzer [24]. Networks of highly-related parasites were created using only monoclonal samples using the R igraph package [28]. Moreover, IBD distribution across the genome a was calculated using isoRelate R package as described by Henden et al*.* [20]. Then the proportion of pairs IBD (XiR,s), where XiR,s is the chi square distribution IBD at each SNP was estimated as described by Henden et al*.* [20]. P-values were calculated and a 5% genome-wide significance threshold was used to differentiate loci with selection signals. Spatial distance between sample pairs was measured both with greater circle distance and road distance to measure isolation by distance using the Mantel test. To assess temporal changes in parasite clustering due to control pressure and identify genetic outliers that are possible imported cases, principal component analysis (PCA) was conducted using within-sample allele frequencies. PCA results with other inputs, including major alleles, did not differ. To further investigate parasite population clustering, t-distributed Stochastic Neighbor Embedding (t-SNE) was calculated using the R package Rtsne. The same loci were extracted from publically available data (n = 909, from the Democratic Republic of the Congo (DRC), Malawi, Tanzania and other regions of Zambia) for PCA analysis to assess the possibility of parasite importation.

### Statistical analysis

All references to an analysis in a ‘package’ indicate the analysis was performed in R software [29]. Where appropriate, all outputs were visualized using the ggplot2 package in R. The Mann–Whitney U test was used to measure differences among two groups and the Kruskal–Wallis test was used to measure differences between more than two groups. A confidence interval for IBD values was calculated as mean IBD ± 1.96 X standard error of IBD, where standard error IBD = standard deviation of IBD/square root of (n) where n = sample size. A P-value of ≤ 0.05 was considered statistically significant.

## Results

**Fig. 2:**
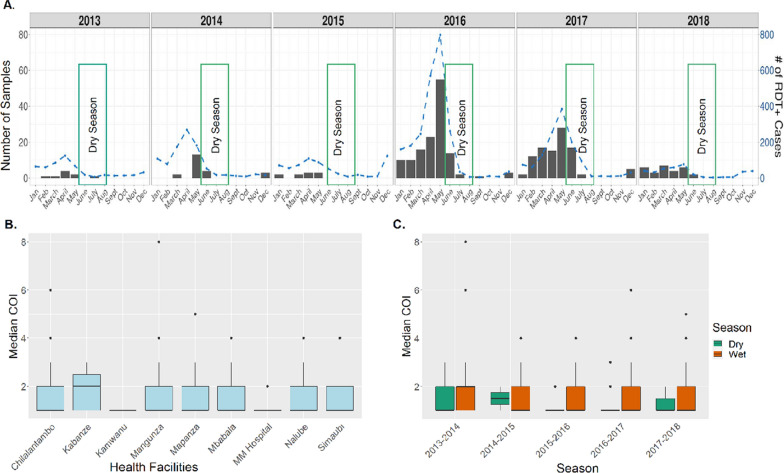
Malaria trends and complexity of infections. **A** Trends of RDT positive cases at the 8 health centres over the course of the study (blue dotted line) and the number of dried blood spot (DBS) samples collected by month over the course of the study (gray bars). Dark green bars show dry seasons (June to August). **B** Spatial heterogeneity of complexity of infections. **C** Seasonal variation of multiplicity of infections. The box- and whisker-plots were generated from the median number clones determined per sample. Boxes indicate the interquartile range, the line indicates the median, and the whiskers show the 95% confidence intervals. Dots indicate any outlier values and colours indicate seasons

The epidemiology of malaria in the Macha Hospital catchment area within Choma District from 2012 to 2018 was seasonal (Fig. 2A). The rainy season occurs from November to May. Malaria cases start to increase around December of each calendar year, peaking around April or May the following year. Cases then decline during the dry season (June to August) with few cases reported from July through November. A total of 441 DBS samples were collected from eight rural health centres and Macha Hospital (Additional file 1: Fig. S1) during the study period and 302 (68%) were successfully genotyped at 1410 positions across the genome (Additional file 1: Fig. S2, Additional file 2: Table S1). These samples are representative of almost all time periods from which the original sample set was collected (Additional file 1: Fig. S3). A total of 84% of samples (n = 255) had less than 10% missing genotype calls across all positions (Additional file 1: Fig. S4). Most successfully genotyped samples were collected from 2016 and 2017, but samples representing all years between 2013 and 2018 were successfully genotyped and reflect the epidemiologic case data (Fig. 2A, Additional file 2: Table S2).

Genetic data generated through MIPs showed signs of both increased transmission in the high transmission season and parasite population connectivity across seasons, both expected in regions that have seasonal trends. Complexity of infection (COI) (Fig. 2B) often tracks with transmission intensity [24, 30, 31] and COI varied at health facilities suggesting spatial heterogeneity of malaria transmission at local scales. Most (67%, n = 202) infections were monogenomic (COI = 1) (Additional file 1: Fig. S5A), as expected in an area with overall low transmission. Median COI estimates were relatively higher during the rainy season, and the lowest average COI estimates were during the dry season, though the difference was not statistically significant (Kruskal–Wallis test, *p* = 0.12). Overall COI was relatively stable over the study period, with no significant declines in recent years despite overall reduced transmission (Additional file 1: Fig. S5B). This suggests there is ongoing asymptomatic transmission outside the symptomatic cases presenting to health centres sampled in this study. This asymptomatic transmission likely contributes to recombination of different distinct clones and maintenance of a diverse genetic pool despite low transmission.

**Fig. 3:**
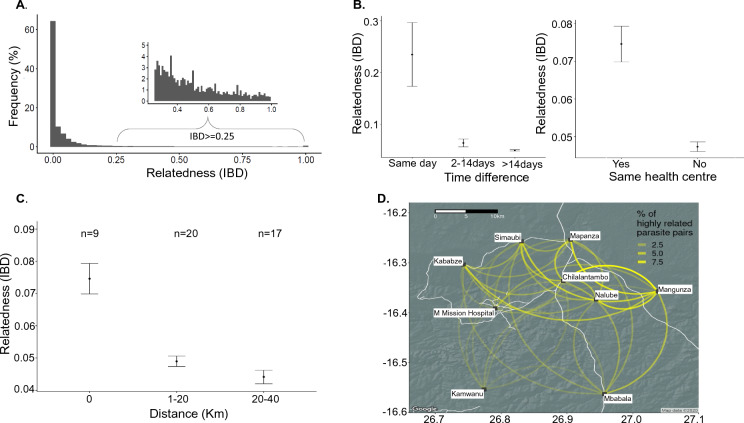
Temporal and spatial patterns in pairwise genetic relatedness. **A** Shows the genetic relatedness between all samples using the *F* statistic. The insert shows the number of samples with high relatedness (IBD ≥ 0.25). **B** Shows that isolates collected on the same day and within the same health centre were more likely to be highly related. **C** Shows a pattern of isolation by increasing geographic distance. **D** Shows the proportion of highly related parasite pairs shared between health centres. The width of the yellow connecting line corresponds to the proportion of shared highly related parasites. Major roads connecting health centres are shown in white

To examine genetic relatedness, we calculated IBD using the inbreeding coefficient *F* between all 45,150 pairs of genomic regions across 1410 loci for 302 samples [24]. Most comparisons had little to no IBD sharing; however, 6% of comparisons had an IBD ≥ 0.25 (half siblings) (Fig. 3A). There was also uneven distribution of IBD segments across the genome (Additional file 1: Fig. S6A) suggesting variation recombination at different genomic regions of parasites due to different selection pressures. Using a 5% cutoff threshold, four chromosomes (1, 5, 11, and 13) were identified to be under selection (Additional file 1: Fig. S6B). We looked at genomic regions near (± 1 kb) loci under selection and found 45 genes including *kelch13* (an artemisinin resistance marker) (Additional file 2: Table S3), suggesting parasites are under drug pressure that warrants close monitoring to prevent the emergence and spread anti-malarials resistance in this low malaria transmission setting.

Parasite pairs from the same health centre were more likely to have higher IBD values (Fig. 3B), with an approximate isolation-by-distance trend observed between different health centres (Fig. 3C). To identify whether transmission hotspots of highly-related infection pairs occurred between health centres, the number of highly-related (IBD ≥ 0.25) infection pairs occurring between two health centres was shown as a proportion of the total number of highly related pairs (Fig. 3D). Health centres connected by major roads (Additional file 1: Fig. S7A) have significantly higher (Mann–Whitney *p* < 0.001) related parasites than those not connected by major roadways (such as Kamwanu) (Additional file 1: Fig. S7B), although geographic proximity could override this pattern (i.e., Nalube). However, all health centres, even those geographically distant or not connected by major roadways, had some highly-related parasite pairs.

**Fig. 4:**
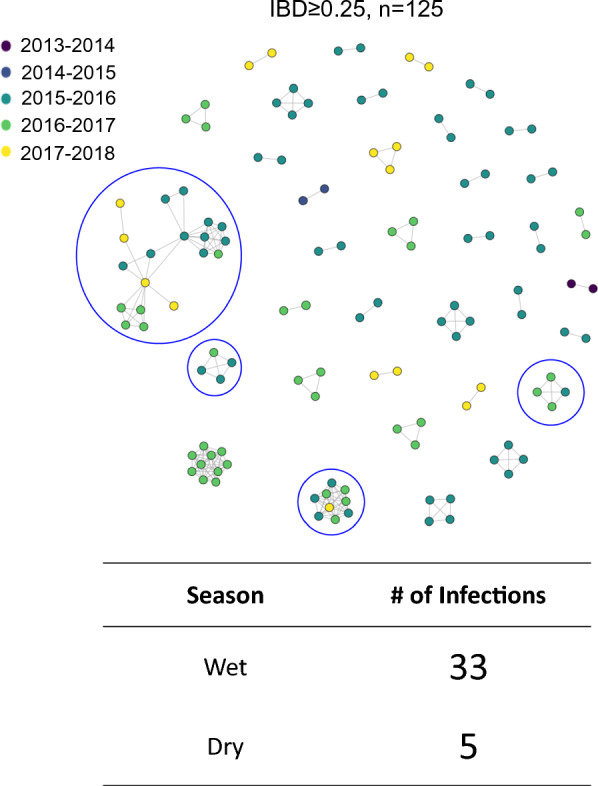
IBD network of monoclonal samples with IBD ≥ 0.25 shows transmission across multiple seasons and through the dry season. Networks spanning multiple seasons are circled in blue. The samples are coloured by the season of collection and only monogenomic samples (COI = 1) are included. Thirty-eight samples were contained in networks spanning multiple seasons, with five representing samples collected in the low transmission dry season. Values indicate IBD cutoff and number of samples (n) above the IBD cutoff

Similar to the observed spatial trends, parasites collected on the same day had higher IBD values than parasites collected further apart in time (Fig. 3B). To investigate temporal patterns of genetic relatedness, networks were built using only monogenomic infections. Interestingly, these networks showed patterns of highly-related (IBD ≥ 0.25) infection pairs across months of the same malaria season and even between malaria seasons using lower IBD threshold (Additional file 1: Fig. S8). Using a cut off of IBD ≥ 0.25, the equivalent of half-siblings or closer relationships, networks of monogenomic infections were identified involving 38 isolates across multiple seasons that also contained parasites sampled during the dry season (n = 5) (Fig. 4), providing evidence that a proportion of the parasite population is maintained through the dry season and contributes to malaria cases the following transmission season.

**Fig. 5:**
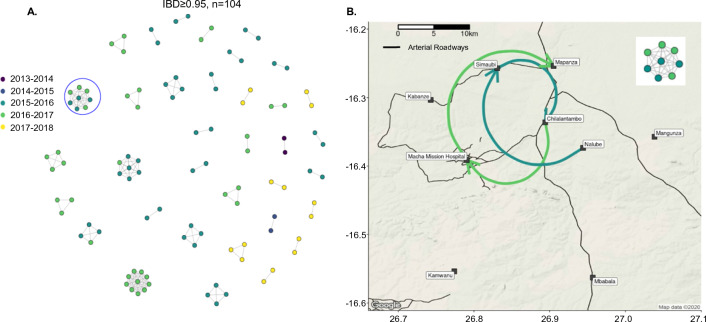
IBD networks of clonal samples (IBD ≥ 0.95) show transmission across seasons and across the study site. **A** Shows networks across seasons using only monoclonal samples. **B** Shows the geographic locations of samples from one of these clusters. These locations are connected by lines coloured to correspond to the season of collection. Arrows on these lines correspond to the order in which the samples were collected. The insert represents the cluster from **A** that is circled in blue and displayed on the map

The largest cluster traverses multiple dry seasons, indicative of long-term propagation with minimal outcrossing in a proportion of parasites. Among clonal samples (IBD ≥ 0.95, n = 104), multi-season networks or those spanning multiple months occurred (Fig. 5A), including three clusters comprising more than seven samples each. These clusters all represent parasites collected from the 2015–2016 and 2016–2017 seasons, the seasons for which most samples were successfully genotyped. Interestingly, these clonal networks showed little spatial clustering with clones appearing across different health centres (Fig. 5B).

While these findings provide evidence that parasites are connected between health centres and are maintained through the dry season, they do not address the potential contribution of imported parasites. To investigate if genetic outliers existed among the sampled parasites, a possible proxy for importation of genetically distinct parasites, a principal component analysis (PCA) was conducted using all parasites (Additional file 1: Fig. S9A). Overall, the first two components explained little of the variation (PC1: 4%, PC2: 3%). However, PC1 and PC2 separated some parasites from all others. Interestingly, these parasites were two of the large IBD ≥ 0.95 clusters described above (Additional file 1: Fig. S9A).

**Fig. 6:**
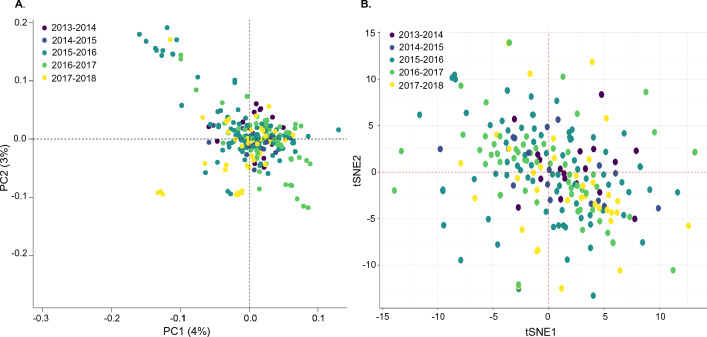
Temporal population structure *P. falciparum* parasite population Choma District, Southern Zambia using PCA (**A**) and t-SNE (**B**). Only single representative samples from each highly related cluster were included in both analyses (see Additional file 1: Fig. S9A for analysis with all samples)

These two outlier clusters of clonal parasites were derived mainly from 2016 and 2017, and were distributed across health centres (Additional file 1: Fig. S10). Moreover, t-SNE analysis also confirmed the lack of substantial population structure by season (Fig. 6B), suggesting the *P. falciparum* population in southern Zambia is ‘panmictic’ with no definitive evidence of importation determined in this study. All results from PCA, t-SNE and IBD analyses suggest high parasite movement and mixing between and within health centres at the district level, with large interconnected clusters of isolates more common across nearby health centres compared to distant health centres—a pattern is consistent with isolation-by-distance. Overall, the result gives some insight into the district or local parasite movement. Connectivity is a key challenge as settings approach pre-elimination and as it helps maintain a diverse genetic pool of parasites and potentially facilitates greater resilience to control pressures.

## Discussion

Genomic analysis using a novel high-density genome-wide genotyping platform showed a detailed snapshot of the transmission dynamics that connect falciparum malaria infections across space and time in the Choma District of southern Zambia from 2012 to 2018, an area of low transmission. Despite the relatively small geographic study region (2000 sq km), IBD analysis was able to capture different genomic signals that reflects low malaria transmission intensity in southern Zambia. Overall, most infections were monogenomic (COI = 1) and networks of identical or highly related parasites persisted across space and time. These were detected in the dry low transmission season followed by “seeding” the parasite population in the next high transmission season (Figs. 4 and 5). These parasites were then transmitted widely across the district, likely due to the relatively small geographic scale. These transmission networks of highly related parasites are consistent with outbreaks that appear to overlay a background parasite population that maintains an overall weak signal of isolation by distance (Fig. 3). Interestingly, two of the clonal networks were among clonal parasites that were more related within the network than the general parasite population, but did not cluster with parasite populations in the northern part of Zambia or other countries sharing the border with Zambia. This fracturing of the parasite population suggests that as countries move to pre-elimination, increasing spatial heterogeneity of malaria transmission is expected with more focal clustering of clonal or highly related parasites. The data also do not suggest importation as a major driver of parasite diversity in the samples analysed, and importation with resultant local spread likely did not lead to these clusters. However, to fully define parasite importation genomic information from contemporary parasite populations outside Choma District, denser sampling of parasites (including asymptomatic parasitaemia), as well as information on human movement patterns are required. Moreover, asymptomatic cases, which are invisible to current surveillance approaches, could also contribute to ongoing endemic transmission and increase diverse parasite pools in the population, leading to parasite recombination in the mosquito which ultimately increases COI within the host. These findings suggest that some genomic metrics (i.e., COI, genetic diversity) have limitations for measuring transmission intensity for samples collected through passive cases detection at health facilities. Thus, active case detection, which captures both asymptomatic and low parasitaemia cases, are recommended to identify correlations between COI and malaria transmission at the community level, although low level parasitaemia can be challenging for parasite genotyping.

Genomic studies of local transmission and patterns of parasite connectivity in low and medium endemicity countries are becoming more common and starting to shed light on the dynamics of malaria parasite movement [3, 15–17]. However, relatively few studies have used genomic approaches to study malaria transmission dynamics and parasite clustering on smaller geographic scales, such as a single district [32]. Detailed work in Senegal has shown similar findings, including persistence of clonal parasites across multiple seasons and evidence of highly related parasites as transmission declines due to control interventions [8, 33, 34]. However, the approaches used in these studies consisted of lower density genotyping, primarily with a 24 single nucleotide polymorphism (SNP) barcode. This study adds to the existing literature as the high-density genotyping allows for the use of IBD to help resolve finer scale parasite clustering and relatedness within the population.

Unlike previous work in this setting [32], IBD was able to evaluate networks of parasites based on the number of outcrossings, showing that networks of half-siblings or more highly related parasites are extensive and persist for longer than networks of clonal parasites (Figs. 4 and 5). In addition, this metric, in combination with epidemiological data, provided insight to define the underlying isolation-by-distance relationship seen in the general parasite population in Choma District as well as the persistence of clonal parasites that escaped control intervention and persisted across seasons. Signals of isolation by distance and parasite population fracturing at finer geographic scales suggest different processes, such as the distribution and variation in utilization of malaria control intervention, topography, road networks, uneven sampling, and sample size variation, that could contribute to this variation. Moreover, in low transmission settings the variability of malaria vectors in local adaptation [35] and vector–parasite compatibility [36] could contribute to the observed genomic signal variation (i.e. from genetically diverse parasite to clonal and related parasite clustering). In addition, as noted previously [37], MIPs provide a cost-effective means of high resolution genotyping of large numbers of parasites compared to whole genome sequencing and is therefore a scalable tool to provide detailed studies of transmission and importation with large numbers of samples. Lastly, within the study region, fluctuations in transmission intensity were detected, as measured by COI, between seasons, suggesting a ramp-up and expansion of parasite populations with each season. COI has previously been shown to be correlated with local transmission intensity [24, 30, 31].

This work demonstrates maintenance of parasite clones through the dry season and increasing parasite clonal transmission leading to population fragmentation in recent years supporting the effectiveness of recent control efforts. Moreover, our findings did not show a significant contribution of imported malaria cases for sustainable malaria transmission in the area. However, there are multiple limitations to identifying imported cases. First, the sampling of only acute cases will limit the ability to define local transmission dynamics and identify importations. The asymptomatic reservoir likely contributes significantly to sustained low level transmission [38]. However, given the low density parasitaemias of asymptomatic infections, genotyping these parasites remain a challenge with any platform. Second, a lack of detailed travel histories from the malaria cases, making it impossible to determine if the genetically unrelated parasites were imported. Lastly, the relatively small sample size prevents us from quantifying the relative burden of importation versus persistent transmission over the dry season on the parasites that dominate in the following high transmission season. This will require further studies with dense sampling of symptomatic and asymptomatic cases with travel histories to address how parasite importation contributes to sustained malaria transmission in areas approaching pre-elimination.

The success of malaria elimination in low transmission regions in Africa will depend on a deeper understanding of transmission dynamics and importation. However, there was evidence of parasite movement and connectivity at small spatial scales at the health facilities level, which helps parasites maintain a diverse genetic pool in low transmission areas, potentially creating a challenge for malaria elimination. As recommended by the WHO, defining imported vs. locally acquired cases is critical for designing and stratifying targeted interventions [39, 40]. This work demonstrates the feasibility of genome-wide approaches to help define the relationships between infections in settings approaching malaria elimination. The genotyping tools and analytical methods for these studies are continually advancing. Although the IBD analysis largely focused on monoclonal samples, significant information likely can be gathered from addressing polygenomic infections.

Preliminary work suggests that these tools will still be effective in studying parasite populations in high transmission settings and patterns of parasite connectivity [24]. In addition, combining human mobility data with these genomic tools will help to better understand both local transmission and source of importation.

## Conclusion

This study leveraged state of the art genomic tools and analytical methods to provide a detailed snapshot of the transmission dynamics of malaria in a region on the cusp of elimination and highlights the feasibility of these methods to inform targeted interventions to achieve and sustain malaria elimination.

## Supplementary Information

**Additional file 1: Figure S1.** Study sites in Macha Region. **Figure S2.** Distribution of retained SNPs across *Plasmodium falciparum* chromosomes. **Figure S3.** Retained samples after filtering. **Figure S4.** Missingness by probe and sample. **Figure S5.** Complexity of infection. **Figure S6.** Distribution of IBD segments and selection signals across the *Plasmodium falciparum* genome. **Figure S7.** Study sites in Macha district and spatial pattern parasite relatedness. **Figure S8.** Network analysis of parasite relatedness across season at varying degrees of relatedness. **Figure S9.** Population structure of *P. falciparum* parasite from Macha and bordering countries. **Figure S10.** Spatio-temporal distribution of two outlier clonal cluster parasites described in **Figure S9A**.

**Additional file 2: Table S1.** List of loci included in genome wide MIP panel and successfully genotyped. **Table S2.** Metadata and complexity infection for 302 successfully sequenced samples genome wide MIP panel -this data used for population genetic analysis. **Table S3.** List of genes with signature of selection among the 302 *P. falciparum* isolates.

## Data Availability

All sequencing data available under Accession no. SAMN29983042–SAMN29983315 at the Sequence Read Archive (SRA) (http://www.ncbi.nlm.nih.gov/sra), and the associated BioProject alias is PRJNA862735. All code was previously submitted to GitHub through prior publications.

## References

[1] Masaninga F, Chanda E, Chanda-Kapata P, Hamainza B, Masendu HT, Kamuliwo M (2013). Review of the malaria epidemiology and trends in Zambia. Asian Pac J Trop Biomed.

[2] Björkman A, Shakely D, Ali AS, Morris U, Mkali H, Abbas AK (2019). From high to low malaria transmission in Zanzibar-challenges and opportunities to achieve elimination. BMC Med.

[3] Tessema S, Wesolowski A, Chen A, Murphy M, Wilheim J, Mupiri A-R (2019). Using parasite genetic and human mobility data to infer local and cross-border malaria connectivity in Southern Africa. Elife.

[4] Fola AA, Nate E, Abby Harrison GL, Barnadas C, Hetzel MW, Iga J (2018). Nationwide genetic surveillance of *Plasmodium vivax* in Papua New Guinea reveals heterogeneous transmission dynamics and routes of migration amongst subdivided populations. Infect Genet Evol.

[5] Ruybal-Pesántez S, Sáenz FE, Deed S, Johnson EK, Larremore DB, Vera-Arias CA (2021). Clinical malaria incidence following an outbreak in Ecuador was predominantly associated with *Plasmodium falciparum* with recombinant variant antigen gene repertoires. MedRxiv.

[6] Patel JC, Taylor SM, Juliao PC, Parobek CM, Janko M, Gonzalez LD (2014). Genetic evidence of importation of drug-resistant *Plasmodium falciparum* to Guatemala from the Democratic Republic of the Congo. Emerg Infect Dis.

[7] Mundagowa PT, Chimberengwa PT (2020). Malaria outbreak investigation in a rural area south of Zimbabwe: a case-control study. Malar J.

[8] Daniels RF, Schaffner SF, Wenger EA, Proctor JL, Chang H-H, Wong W (2015). Modeling malaria genomics reveals transmission decline and rebound in Senegal. Proc Natl Acad Sci USA.

[9] Nkhoma SC, Nair S, Al-Saai S, Ashley E, McGready R, Phyo AP (2013). Population genetic correlates of declining transmission in a human pathogen. Mol Ecol.

[10] Branch OH, Sutton PL, Barnes C, Castro JC, Hussin J, Awadalla P (2011). *Plasmodium falciparum* genetic diversity maintained and amplified over 5 years of a low transmission endemic in the Peruvian Amazon. Mol Biol Evol.

[11] Roh ME, Tessema SK, Murphy M, Nhlabathi N, Mkhonta N, Vilakati S (2019). High genetic diversity of *Plasmodium falciparum* in the low-transmission setting of the Kingdom of Eswatini. J Infect Dis.

[12] Raman J, Gast L, Balawanth R, Tessema S, Brooke B, Maharaj R (2020). High levels of imported asymptomatic malaria but limited local transmission in KwaZulu-Natal, a South African malaria-endemic province nearing malaria elimination. Malar J.

[13] Obaldia N, Baro NK, Calzada JE, Santamaria AM, Daniels R, Wong W (2015). Clonal outbreak of *Plasmodium falciparum* infection in eastern Panama. J Infect Dis.

[14] Chenet SM, Schneider KA, Villegas L, Escalante AA (2012). Local population structure of *Plasmodium*: impact on malaria control and elimination. Malar J.

[15] Noviyanti R, Coutrier F, Utami RAS, Trimarsanto H, Tirta YK, Trianty L (2015). Contrasting transmission dynamics of co-endemic *Plasmodium vivax* and *P. falciparum*: implications for malaria control and elimination. PLoS Negl Trop Dis.

[16] Kattenberg JH, Razook Z, Keo R, Koepfli C, Jennison C, Lautu-Gumal D (2020). Monitoring *Plasmodium falciparum* and *Plasmodium vivax* using microsatellite markers indicates limited changes in population structure after substantial transmission decline in Papua New Guinea. Mol Ecol.

[17] Gwarinda HB, Tessema SK, Raman J, Greenhouse B, Birkholtz L-M (2021). Parasite genetic diversity reflects continued residual malaria transmission in Vhembe District, a hotspot in the Limpopo Province of South Africa. Malar J.

[18] Kobayashi T, Jain A, Liang L, Obiero JM, Hamapumbu H, Stevenson JC (2019). Distinct antibody signatures associated with different malaria transmission intensities in Zambia and Zimbabwe. mSphere.

[19] Taylor AR, Schaffner SF, Cerqueira GC, Nkhoma SC, Anderson TJC, Sriprawat K (2017). Quantifying connectivity between local *Plasmodium falciparum* malaria parasite populations using identity by descent. PLoS Genet.

[20] Henden L, Lee S, Mueller I, Barry A, Bahlo M (2018). Identity-by-descent analyses for measuring population dynamics and selection in recombining pathogens. PLoS Genet.

[21] Schaffner SF, Taylor AR, Wong W, Wirth DF, Neafsey DE (2018). hmmIBD: software to infer pairwise identity by descent between haploid genotypes. Malar J.

[22] Topazian HM, Gumbo A, Puerto-Meredith S, Njiko R, Mwanza A, Kayange M (2020). Asymptomatic *Plasmodium falciparum* malaria prevalence among adolescents and adults in Malawi, 2015–2016. Sci Rep.

[23] Pickard AL, Wongsrichanalai C, Purfield A, Kamwendo D, Emery K, Zalewski C (2003). Resistance to antimalarials in Southeast Asia and genetic polymorphisms in pfmdr1. Antimicrob Agents Chemother.

[24] Verity R, Aydemir O, Brazeau NF, Watson OJ, Hathaway NJ, Mwandagalirwa MK (2020). The impact of antimalarial resistance on the genetic structure of *Plasmodium falciparum* in the DRC. Nat Commun.

[25] Moser KA, Madebe RA, Aydemir O, Chiduo MG, Mandara CI, Rumisha SF (2021). Describing the current status of *Plasmodium falciparum* population structure and drug resistance within mainland Tanzania using molecular inversion probes. Mol Ecol.

[26] Garrison E, Marth G. Haplotype-based variant detection from short-read sequencing. arXiv [q-bio.GN]. 2012. http://arxiv.org/abs/1207.3907.

[27] Chang HH, Worby CJ, Yeka A, Nankabirwa J, Kamya MR, Staedke SG (2017). THE REAL McCOIL: a method for the concurrent estimation of the complexity of infection and SNP allele frequency for malaria parasites. PLoS Comput Biol.

[28] Csárdi G, Nepusz T. The igraph software package for complex network research. 2006. https://www.semanticscholar.org/paper/1d2744b83519657f5f2610698a8ddd177ced4f5c. Accessed 17 Aug 2020.

[29] Ripley BD. The R project in statistical computing. MSOR connect. Educational Development Unit, University of Greenwich; 2001;1:23–5.

[30] Hendry JA, Kwiatkowski D, McVean G (2021). Elucidating relationships between *P. falciparum* prevalence and measures of genetic diversity with a combined genetic-epidemiological model of malaria. PLoS Comput Biol.

[31] Watson OJ, Okell LC, Hellewell J, Slater HC, Unwin HJT, Omedo I (2021). Evaluating the performance of malaria genetics for inferring changes in transmission intensity using transmission modeling. Mol Biol Evol.

[32] Searle KM, Katowa B, Musonda M, Pringle JC, Hamapumbu H, Matoba J (2020). Sustained malaria transmission despite reactive screen-and-treat in a low-transmission area of Southern Zambia. Am J Trop Med Hyg.

[33] Daniels RF, Schaffner SF, Dieye Y, Dieng G, Hainsworth M, Fall FB (2020). Genetic evidence for imported malaria and local transmission in Richard Toll, Senegal. Malar J.

[34] Sy M, Deme A, Warren JL, Daniels RF, Dieye B, Ndiaye PI (2022). *Plasmodium falciparum* genomic surveillance reveals spatial and temporal trends, association of genetic and physical distance, and household clustering. Sci Rep.

[35] Joy DA, Gonzalez-Ceron L, Carlton JM, Gueye A, Fay M, McCutchan TF (2008). Local adaptation and vector-mediated population structure in *Plasmodium vivax* malaria. Mol Biol Evol.

[36] Su X-Z, Zhang C, Joy DA (2020). Host-malaria parasite interactions and impacts on mutual evolution. Front Cell Infect Microbiol.

[37] Aydemir O, Janko M, Hathaway NJ, Verity R, Mwandagalirwa MK, Tshefu AK (2018). Drug-resistance and population structure of *Plasmodium falciparum* across the Democratic Republic of Congo using high-throughput molecular inversion probes. J Infect Dis.

[38] Lin JT, Saunders DL, Meshnick SR (2014). The role of submicroscopic parasitemia in malaria transmission: what is the evidence?. Trends Parasitol.

[39] WHO. A framework for malaria elimination. Geneva: World Health Organization; 2017. https://apps.who.int/iris/bitstream/handle/10665/254761/9789241511988-eng.pdf. Accessed 14 Mar 2021.

[40] Marshall JM, Bennett A, Kiware SS, Sturrock HJW (2016). The hitchhiking parasite: why human movement matters to malaria transmission and what we can do about it. Trends Parasitol.

