# Supplementary material for: Temporal and spatial analysis of Plasmodium falciparum genomics reveals patterns of parasite connectivity in a low-transmission district in Southern Province, Zambia
Source: Malar J. 2023 Jul 7;22:208. doi: 10.1186/s12936-023-04637-9 (PMC10327325; doi:10.1186/s12936-023-04637-9)
Supplement: Supplementary file 1 — Additional file 1: Figure S1. Study sites in Macha Region. Figure S2. Distribution of retained SNPs across Plasmodium falciparum chromosomes. Figure S3. Retained samples after filtering. Figure S4. Missingness by probe and sample. Figure S5. Complexity of infection. Figure S6. Distribution of IBD segments and selection signals across the Plasmodium falciparum genome. Figure S7. Study sites in Macha district and spatial pattern parasite relatedness. Figure S8. Network analysis of parasite relatedness across season at varying degrees of relatedness. Figure S9. Population structure of P. falciparum parasite from Macha and bordering countries. Figure S10. Spatio-temporal distribution of two outlier clonal cluster parasites described in Figure S9A. [file 12936_2023_4637_MOESM1_ESM.doc]

**Supplemental Material for “**Temporal and spatial analysis of *Plasmodium falciparum* genomics reveals patterns of parasite connectivity in a low-transmission district in Southern Province, Zambia”

Abebe A. Fola^1^ *, Kara A. Moser^2^, Ozkan Aydemir^1^, Chris Hennelly^2^, Tamaki Kobayashi^3^, Timothy Shields^3^, Harry Hamapumbu^4^, Michael Musonda^4^, Ben Katowa^4^, Japhet Matoba^4^, Jennifer C. Stevenson^4^, Douglas E. Norris^5^, Philip E. Thuma^4^, Amy Wesolowski^3^, William J. Moss^3,5^, Jeffrey A. Bailey^1,+^, Jonathan J. Juliano^2,6,7,8,+^ on behalf of the Southern and Central Africa International Center of Excellence for Malaria Research (ICEMR)

^1^ Department of Pathology and Laboratory Medicine, Brown University, RI, USA, 02906

^2^ Institute for Global Health and Infectious Diseases, University of North Carolina Chapel Hill, NC, USA, 27599

^3^ Department of Epidemiology, Johns Hopkins Bloomberg School of Public Health, Baltimore, MD, USA, 21205

^4^ Macha Research Trust, Choma District, Zambia

^5^ Department of Molecular Microbiology and Immunology, The Johns Hopkins Malaria Research Institute, Johns Hopkins Bloomberg School of Public Health, Baltimore, MD, USA, 21205

^6^ Division of Infectious Diseases, School of Medicine, University of North Carolina Chapel Hill, Chapel Hill, NC 27599

^7^ Department of Epidemiology, Gillings School of Global Public Health, University of North Carolina Chapel Hill, Chapel Hill, NC, USA, 27599

^8^ Curriculum in Genetics and Molecular Biology, School of Medicine, University of North Carolina Chapel Hill, Chapel Hill, NC 27599

*: Corresponding Author

Abebe A. Fola

55 Claverick Street.

RM#314A

Department of Pathology and Laboratory Medicine

Brown University

[abebe_fola@brown.edu](mailto:abebe_fola@brown.edu)

7652378643

+: Co-senior author

Key Words: *Plasmodium falciparum*, transmission, Zambia, genomics

**ADDITIONAL FILE 1**

**ADDITIONAL FIGURES**


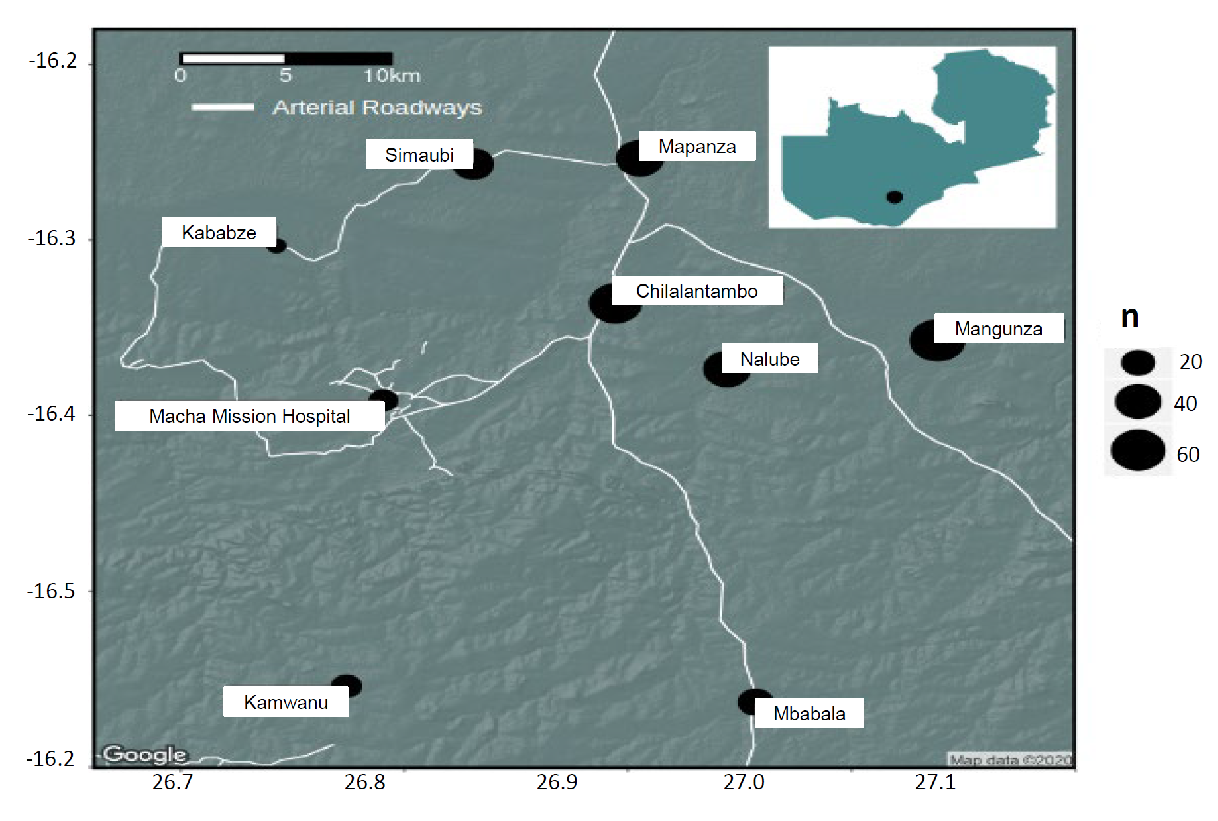


**Fig. S1. Study sites in Macha Region.** The map shows locations of the health centres in the district with the size of the mark representative of the number of samples collected at that clinic.


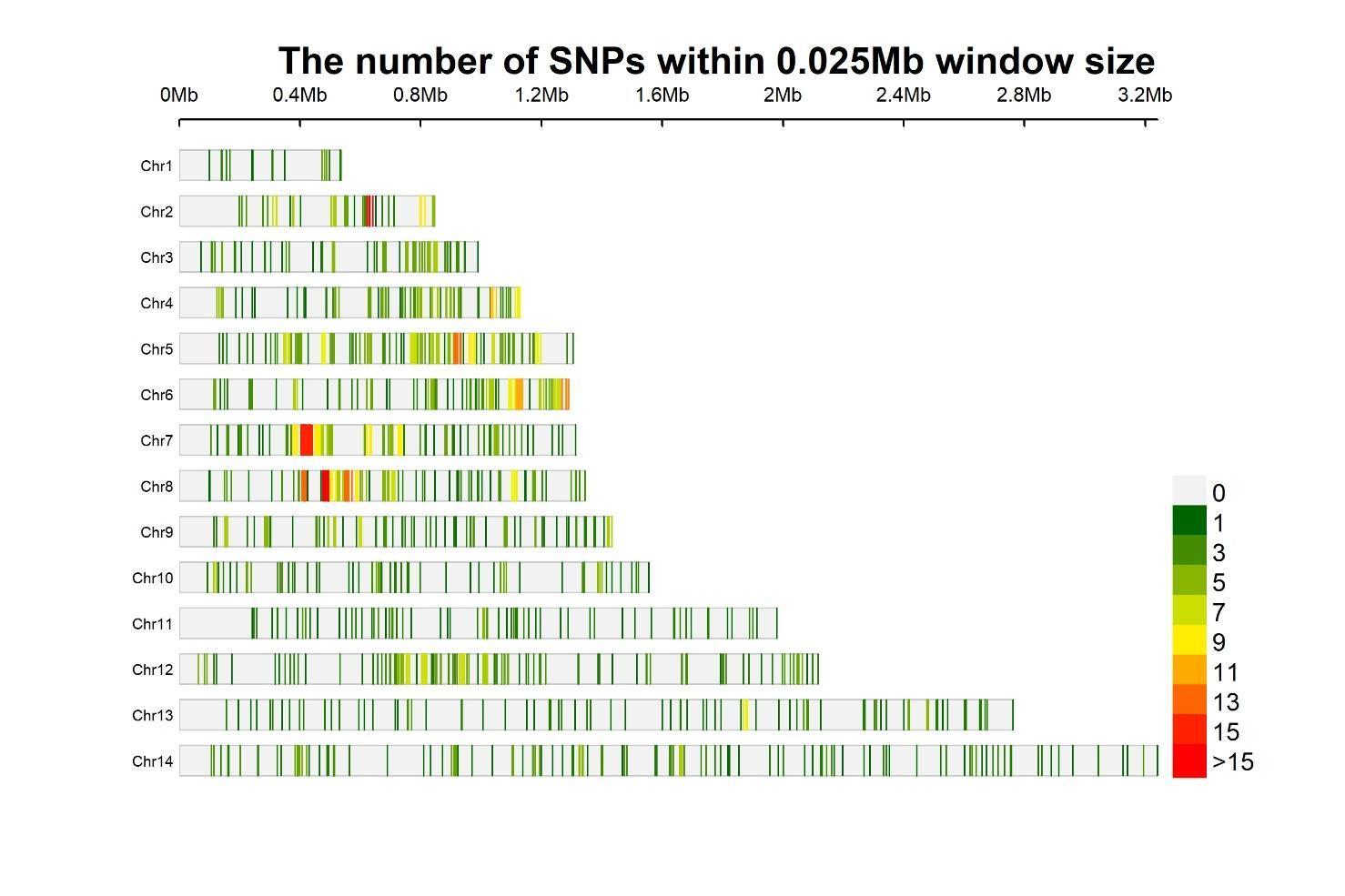


**Fig. S2. Distribution of retained SNPs across *Plasmodium falciparum* chromosomes.** The plot shows distribution of 1410 retained high quality biallelic SNPs across the 14 *P. falciparum* chromosomes within 0.025 Mb window size. Colour coded from light gray for masked regions with no SNPs to red for regions containing high number SNPs per chromosome.


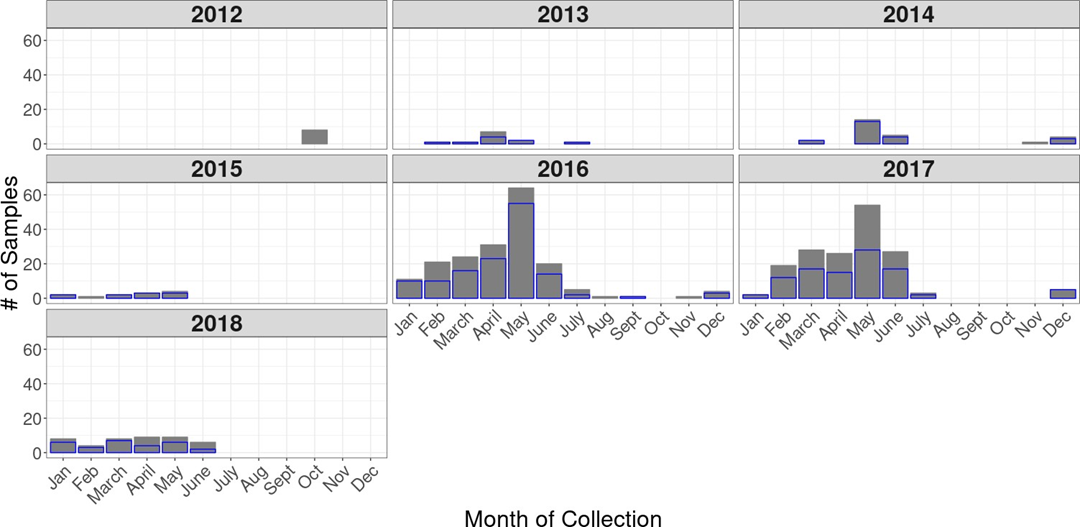


**Fig. S3. Retained samples after filtering.** Total number of samples collected (in gray) are tallied by year and month of collection. Number of samples retained for analysis after filtering are shown in blue. No samples from 2012 were used for downstream analyses.


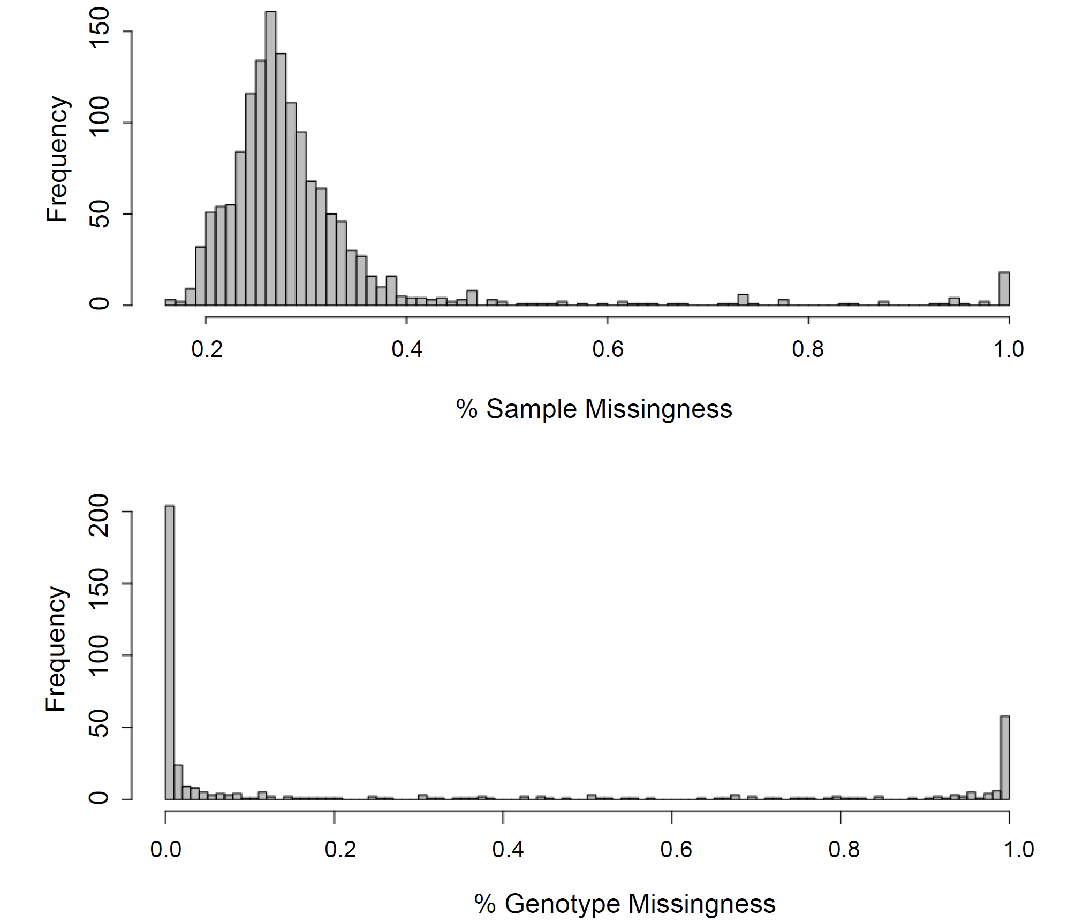


**Fig. S4. Missingness by probe and sample.** Distribution of missingness across each individual targeted MIP position (top), and distribution of missingness across samples (bottom).

#
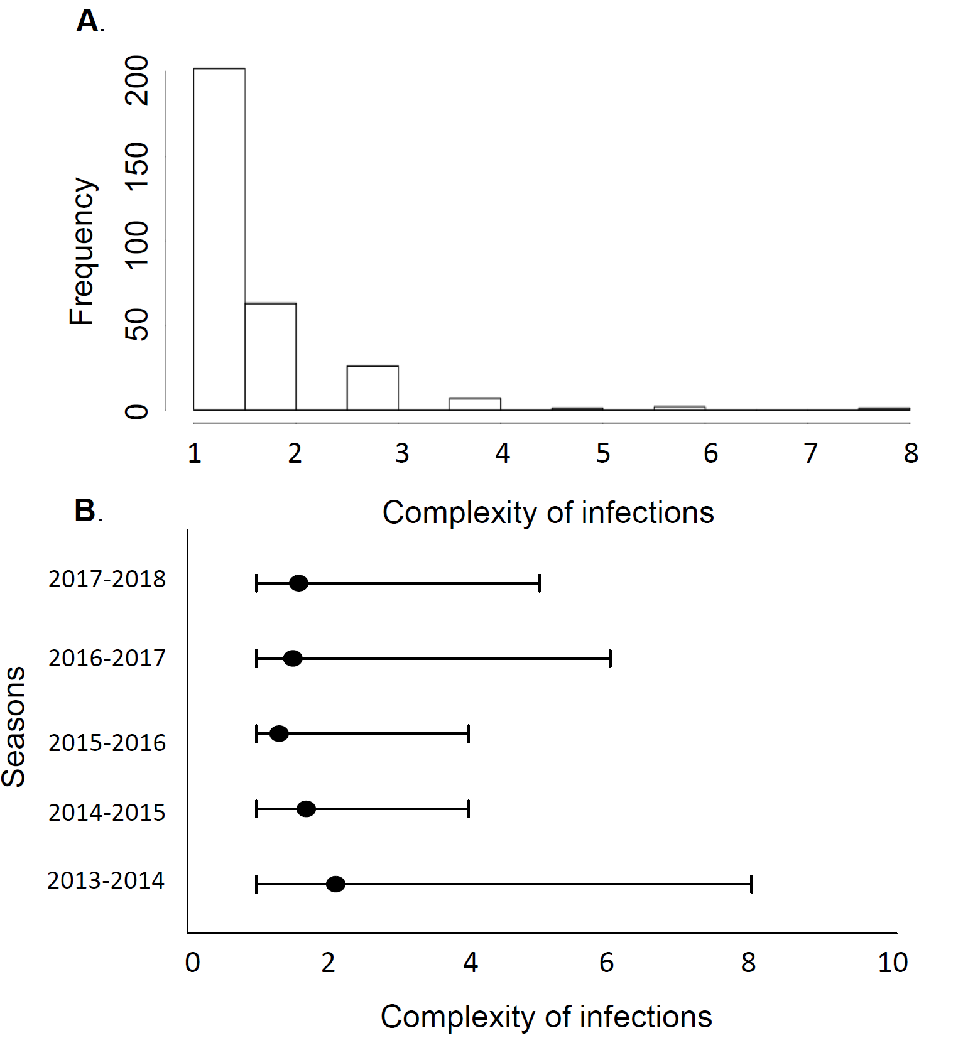


**Fig. S5. Complexity of infection (COI)**. **A)** Distribution of COI estimates from 302 genotyped infections. **B)** Temporal trend of COI, the value indicates median and ranges. Highest number of clones were detected during 2013-2014 where malaria transmission was relatively high.


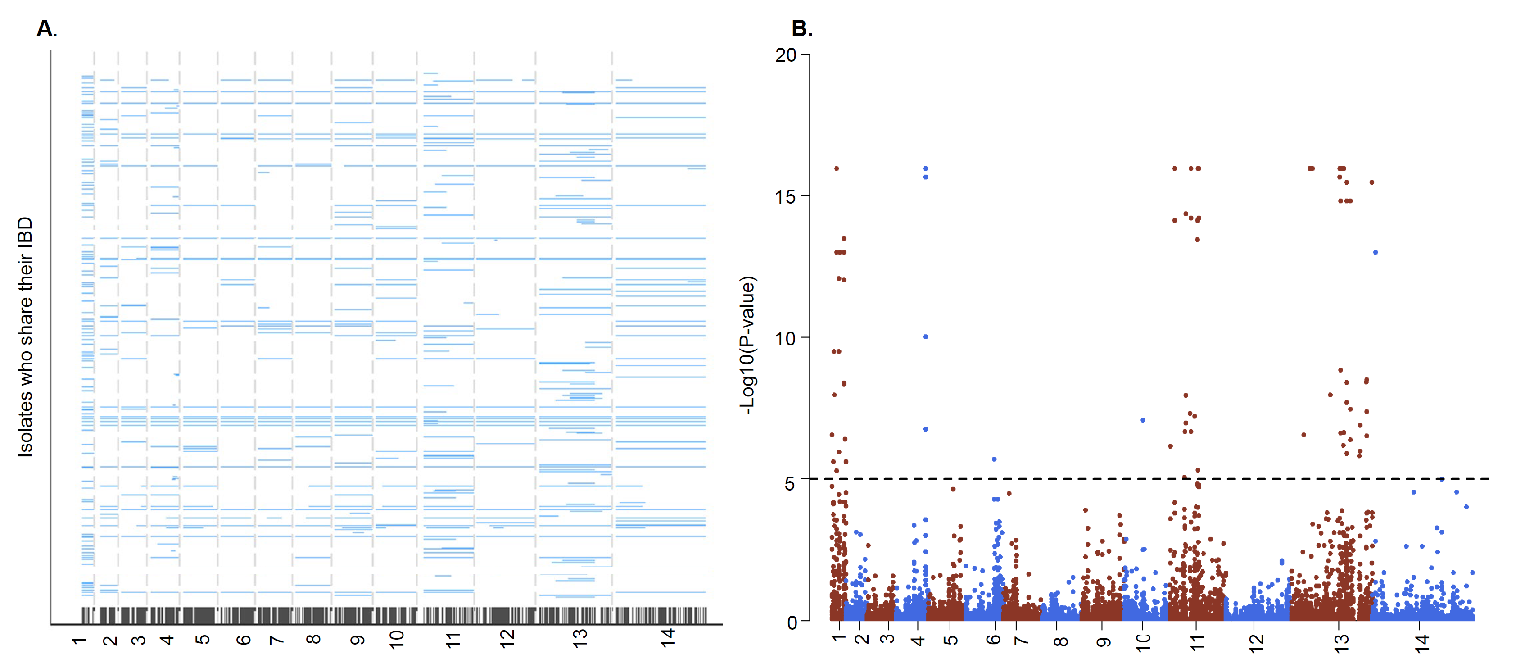


**Fig. S6. Distribution of IBD segments and selection signals across the *Plasmodium falciparum* genome**. **A)** Distribution of IBD segments across the *P. falciparum* genome. Blue colored lines indicate size IBD segment, and black bar at bottom line of the graph shows SNP density. Isolates pairs who are highly related have many IBD segments spanning a large amount of the genome. **B)** Signature of positive selection across the *P. falciparum* genome. Each circled dot represents a SNP. Dashed horizontal black line represents a 5% significance threshold.


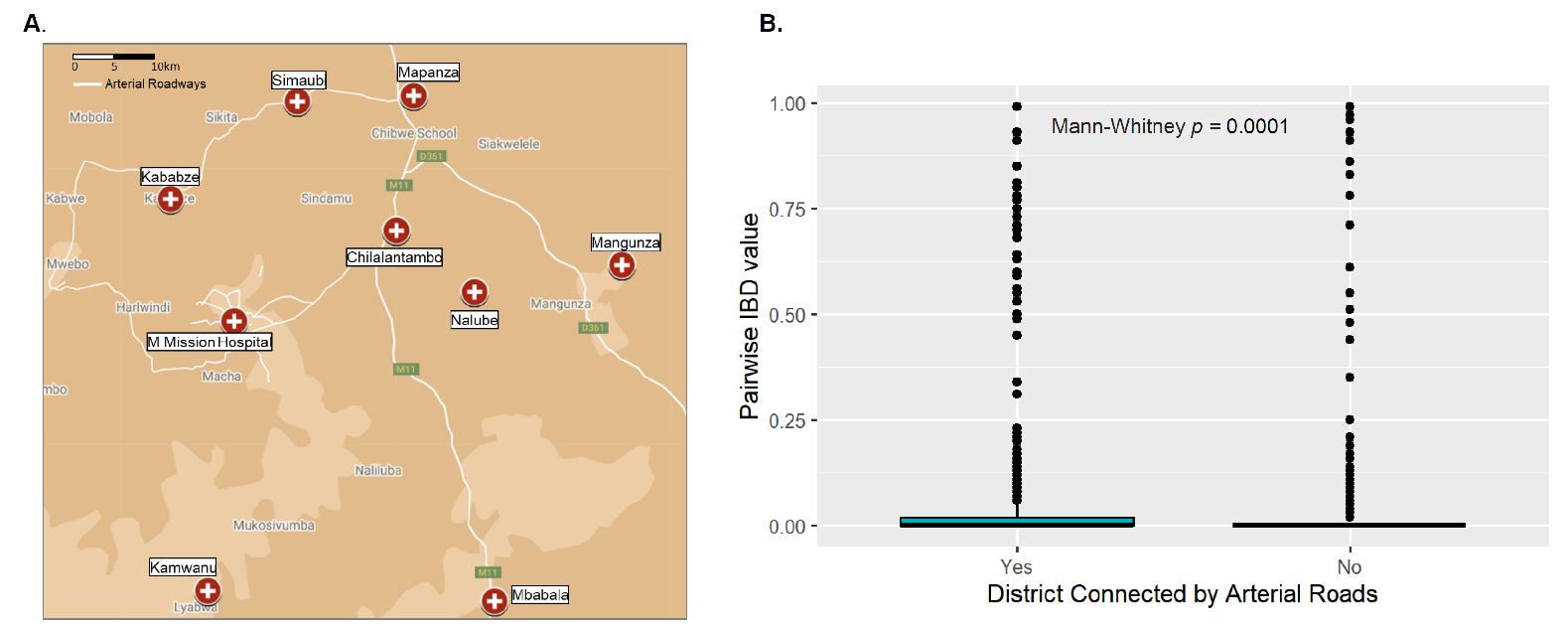


**Fig. S7. Study sites in Macha district and spatial pattern parasite relatedness.** **A)** Map shows study sites (health facilities (+)) included in this study and arterial connecting roads indicated in white line. From nine health facilities three (Kamwanu, Mangunza and Nalube) are not connected by arterial roads. **B)** Plot shows the pairwise IBD sharing between health facilities connected or not connected by arterial roads. Boxes indicate the interquartile range, the line indicates the median, the whiskers show the 95% confidence intervals, and black dots show outlier values*.* The value at the top of the plot indicates *p-value*.


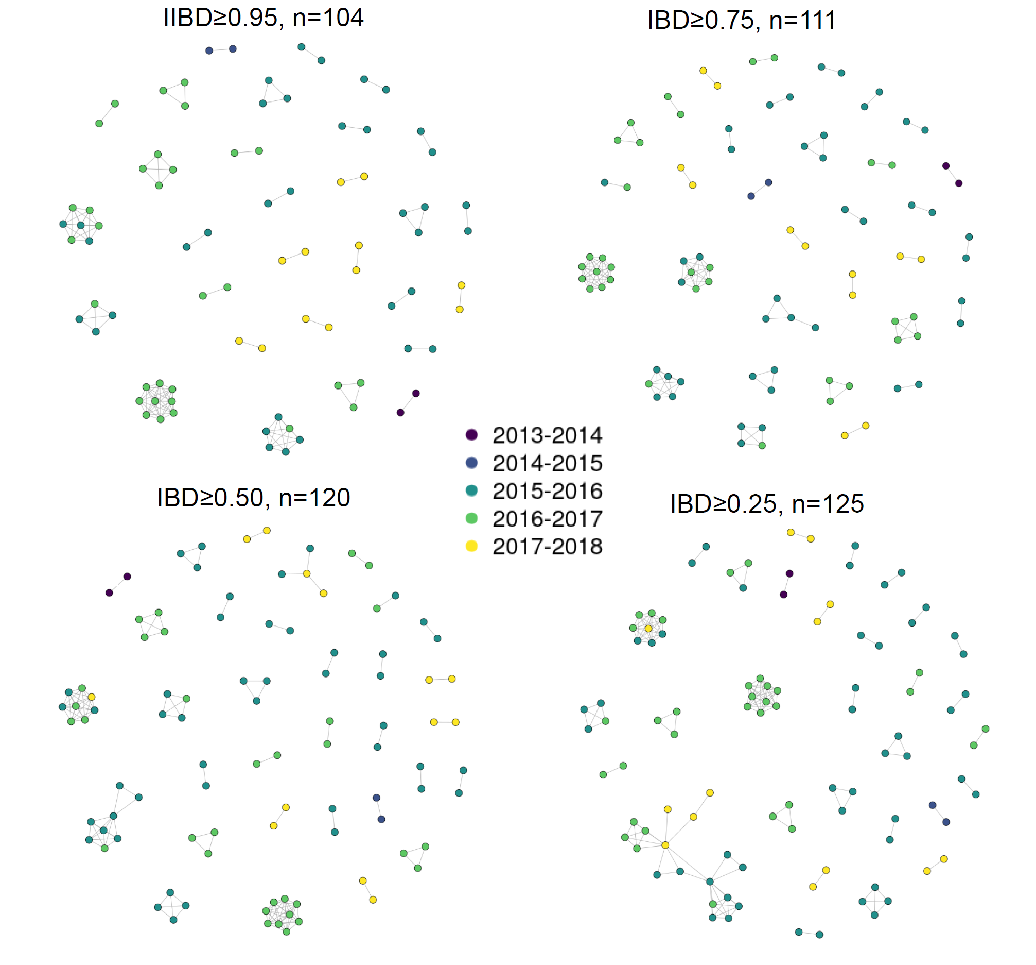


**Fig. S8. Network analysis of parasite relatedness across season at varying degrees of relatedness.** Networks of monoclonal (COI=1) samples were generated at varying levels of genetic relatedness. The season in which a sample was collected is designated by the colour of the circle. As the level of genetic relatedness decreases, increasingly complex networks begin to form and extend over multiple seasons. Values indicate IBD cutoff and number of samples (n) in each network.


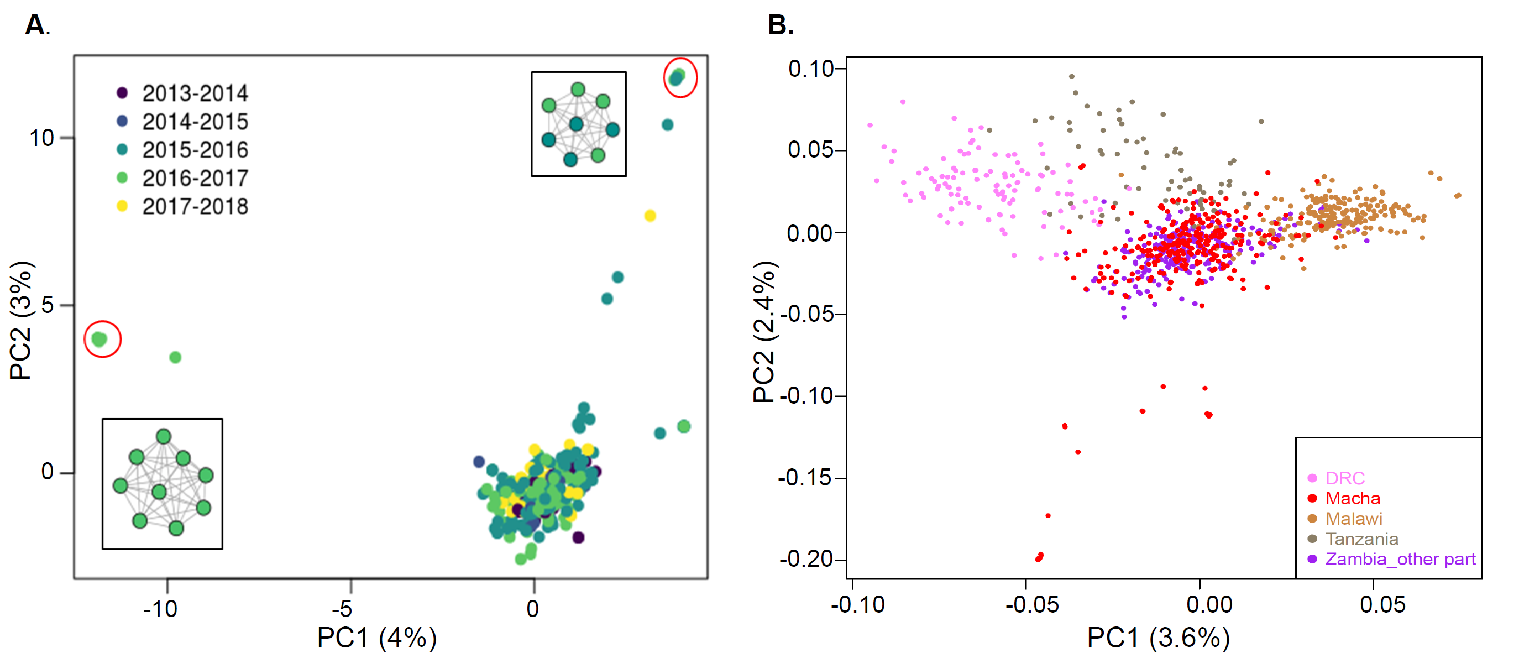


**Fig. S9. Population structure of *P. falciparum* parasite from Macha and bordering countries. A)** The PCA components 1 and 2 of all isolates show several parasite samples that appear genetically different than most samples in the study. Two clusters (circled in red) represent two of the networks of clonal isolates (inserts) previously identified (**Fig. 5**). **B)** Principal component analysis (PCA) of *P. falciparum* populations from the study area in Choma District, Zambia and bordering countries shows outlier samples do not cluster with another parasite population. PCA analysis was done using ‘SNPRelate’ function in R software and plots show the two first principal components out of ten retained PCA.


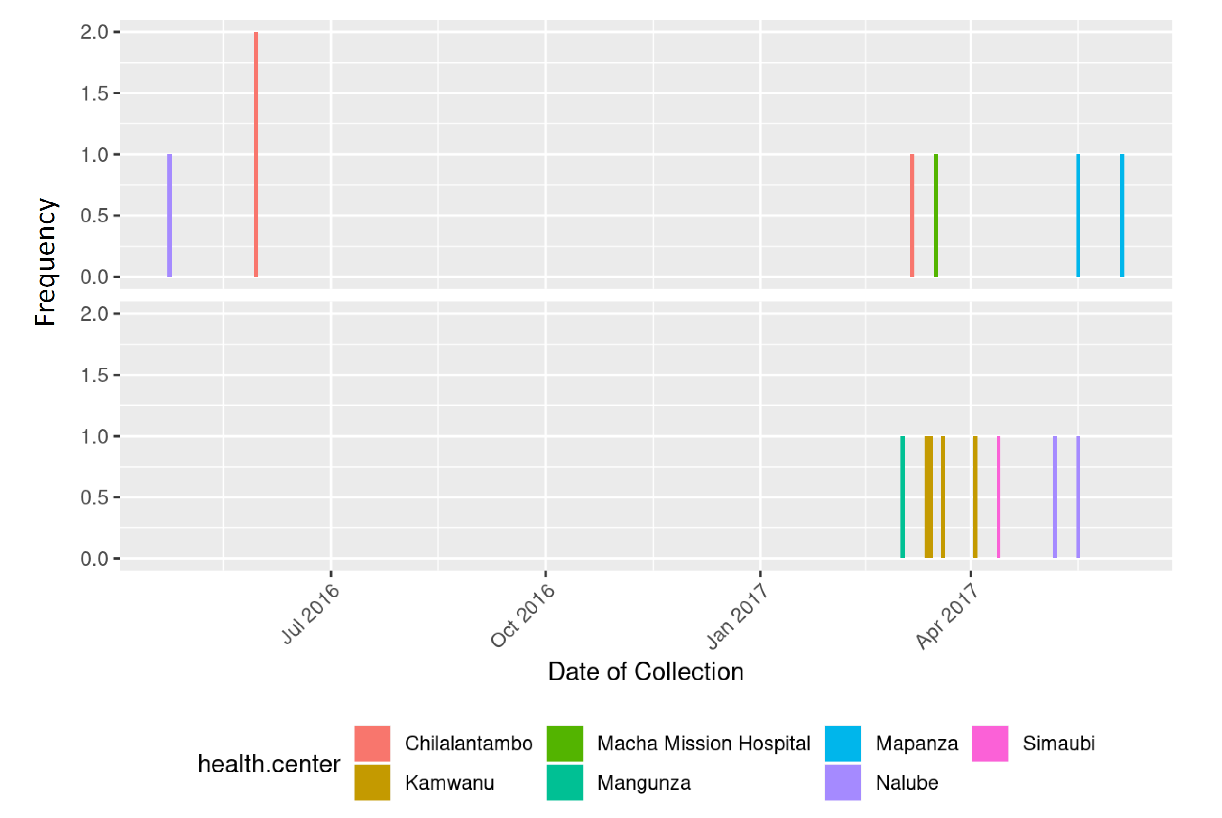


**Fig. S10.** **Spatio-temporal distribution of two outlier clonal cluster parasites described in Fig. S9A.**

**ADDITIONAL TABLES**

*Additional tables are compiled into a single file for ease of viewing.*

**Table S1.** List of loci included in genome wide MIP panel and successfully genotyped.

**Table S2.** Metadata and complexity infection for 302 successfully sequenced samples genome wide MIP panel – this data used for population genetic analysis.

**Table S3.** List of genes with signature of selection among the 302 *P. falciparum* isolates.
